# Is Excessive Polypharmacy a Transient or Persistent Phenomenon? A Nationwide Cohort Study in Taiwan

**DOI:** 10.3389/fphar.2018.00120

**Published:** 2018-02-20

**Authors:** Yi-Jen Wang, Shu-Chiung Chiang, Pei-Chen Lee, Yu-Chun Chen, Li-Fang Chou, Yueh-Ching Chou, Tzeng-Ji Chen

**Affiliations:** ^1^Department of Family Medicine, Taipei Veterans General Hospital, Taipei, Taiwan; ^2^Institute of Hospital and Health Care Administration, National Yang-Ming University, Taipei, Taiwan; ^3^Department of Financial Engineering and Actuarial Mathematics, Soochow University, Taipei, Taiwan; ^4^Department of Pharmacy, Taipei Veterans General Hospital, Taipei, Taiwan; ^5^School of Medicine, National Yang-Ming University, Taipei, Taiwan; ^6^Department of Public Finance, National Chengchi University, Taipei, Taiwan

**Keywords:** polypharmacy, medication management, drug prescriptions, drug utilization, national health programs

## Abstract

**Objectives:** Target populations with persistent polypharmacy should be identified prior to implementing strategies against inappropriate medication use, yet limited information regarding such populations is available. The main objectives were to explore the trends of excessive polypharmacy, whether transient or persistent, at the individual level. The secondary objectives were to identify the factors associated with persistently excessive polypharmacy and to estimate the probabilities for repeatedly excessive polypharmacy.

**Methods:** Retrospective cohort analyses of excessive polypharmacy, defined as prescription of ≥ 10 medicines at an ambulatory visit, from 2001 to 2013 were conducted using a nationally representative claims database in Taiwan. Survival analyses with log-rank test of adult patients with first-time excessive polypharmacy were conducted to predict the probabilities, stratified by age and sex, of having repeatedly excessive polypharmacy.

**Results:** During the study period, excessive polypharmacy occurred in 5.4% of patients for the first time. Among them, 63.9% had repeatedly excessive polypharmacy and the probabilities were higher in men and old people. Men versus women, and old versus middle-aged and young people had shorter median excessive polypharmacy-free times (9.4 vs. 5.5 months, 5.3 vs. 10.1 and 35.0 months, both *p* < 0.001). Overall, the probabilities of having no repeatedly excessive polypharmacy within 3 months, 6 months, and 1 year were 59.9, 53.6, and 48.1%, respectively.

**Conclusion:** Although male and old patients were more likely to have persistently excessive polypharmacy, most cases of excessive polypharmacy were transient or did not re-appear in the short run. Systemic deprescribing measures should be tailored to at-risk groups.

## Introduction

Polypharmacy, usually conceptualized as concurrent use of multiple medications, has raised concerns in recent decades. Evidence has supported the association of polypharmacy with various adverse outcomes, including increased risks of inappropriate prescribing (Fialova and Onder, 2009), drug-drug interactions (Delafuente, 2003), adverse drug reactions, non-adherence, falls, functional decline, hospitalization, and mortality among patients, especially the elderly (Maher et al., 2014). Specifically, long-term use of multiple medications has been found to occur more frequently in cases of adverse drug reactions such as acute renal failure (Chang et al., 2012). The incidence of polypharmacy among patients soars with increasing age (particularly among those more than 65 years old), as do the number of chronic health conditions (Payne et al., 2014) and the number of prescribing physicians (Delafuente, 2003; Green et al., 2007). The number of concurrent medications taken per person most commonly used to define polypharmacy is five, while the cutoff value ranges from two to eleven (Masnoon et al., 2017). In some studies, concomitant use of ten medications or more has been defined as “hyperpolypharmacy,” “excessive polypharmacy,” or “severe polypharmacy" (Masnoon et al., 2017). The more medications a patient takes, the higher risk he or she develops adverse drug events (Green et al., 2007). To address polypharmacy, evidence-based interventions for improving medication management are crucial.

A Cochrane systematic review published in 2015 demonstrated that multi-faceted pharmaceutical care approaches or computerized decision support could reduce inappropriate prescribing to old people with polypharmacy (Cooper et al., 2015). Validated tools (such as the Medication Appropriateness Index (Hanlon et al., 1992), Beers’ criteria (By the American Geriatrics Society 2015 Beers Criteria Update Expert Panel, 2015), Screening Tool of Older Person’s Prescriptions (STOPP)/Screening Tool to Alert doctors to Right Treatment (START) (O’Mahony et al., 2015) were used for the identification of inappropriate medication among elderly patients. The reliability of the conclusions of the review, however, was limited by the small sample sizes of the included studies. “Deprescribing”, another measure aimed at appropriate prescribing, first consists of the careful review of a patient’s medication history, the identification of inappropriate medications, the negotiation with patients about deprescribing plan to discontinue drugs, the switching of drugs or reducing of dosages, the regular review and monitoring of treatment plan, and supporting patients (Woodward, 2003), and was subsequently defined as medication withdrawal in the elderly (Iyer et al., 2008) and then as the cessation of long-term therapy (Le Couteur et al., 2011). Although the deprescribing process had been reported to be effective in improving medication adherence while reducing financial costs (Reeve and Wiese, 2014) and adverse drug reactions (Woodward, 2003), evidence derived from large-scale trials is still lacking. To date, there is a lack of standardization regarding the optimal intervention in cases of polypharmacy. Arguments exist between those taking the view that “less medication is more” (Scott et al., 2015) and those who view polypharmacy as a necessary evil (Wise, 2013). In fact, several medical guidelines [e.g., those for chronic heart failure (Yancy et al., 2017)] require treatment with multiple medications to achieve the optimal clinical effect. As such, it can be argued that appropriate polypharmacy, that is, when medicines are prescribed according to best evidence and the utilization of them has been optimized, could extend life expectancy and maintain quality of life (Duerden et al., 2013). However, most studies related to polypharmacy management have focused on reducing the number of medications instead of on the identification of appropriate polypharmacy or inappropriate underprescribing (Cooper et al., 2015).

Polypharmacy exists in the general community (Fried et al., 2014), long-term care facilities, outpatients, and inpatients (Maher et al., 2014), and people with free healthcare coverage have been found to gave a greater risk of polypharmacy than those without free health care (Richardson et al., 2014). Such drug prescription/utilization patterns are prevalent in Taiwan and generate great burdens in terms of healthcare financing under the current National Health Insurance (NHI) program, which offers universal coverage to beneficiaries. One recent study found that 81.0% of disabled elderly Taiwanese received five or more medications per prescription, that 38.1% received ten or more medications per prescription, and that 32.5% had persistently received five or more medications per prescription (≥181 days) within 1 year (Chan et al., 2009). In 2012, the Ministry of Health and Welfare in Taiwan announced that the “ratio of prescriptions containing more than ten drug items over all prescriptions” during ambulatory visits would be viewed as one of the healthcare quality indicators in the “Act of Openness of Healthcare Quality Information within the NHI.” The policy contains implicit coercion with respect to reducing the number of medications prescribed to all beneficiaries. In this context, it is necessary to identify those people who constitute groups at high risk for persistent and inappropriate polypharmacy before undertaking the deprescribing of medications (Bushardt et al., 2008). However, a lack of evidence regarding the characteristics and patterns of patients with inappropriate polypharmacy has contributed to universal application of the policy to beneficiaries without sufficient differentiation.

Past studies have mostly focused on the negative effects of inappropriate medication use or the positive health outcomes resulting from medication withdrawal (Reeve et al., 2014). Some studies conducted in other countries have specified the target population for medication cessation. A longitudinal study in the Netherlands found that the initial number of long-term drugs, age, diabetes, coronary ischemic diseases, and hypertension were strong predictors for the development of polypharmacy among the elderly (Veehof et al., 2000). There is, however, a relative scarcity of studies that have examined the occurrence of polypharmacy and its association with time on individual levels. In this study, we examined excessive polypharmacy, defined as ten or more medications per prescription per ambulatory care visit, longitudinally at the individual level from a nationwide perspective. The main objectives were to explore the type of excessive polypharmacy, that is, transient or persistent, seen among ambulatory patients with polypharmacy at healthcare facilities of all levels, from the point of the initial prescription indicating excessive polypharmacy until the end of the observation period. The secondary objectives were to identify the risk factors of and to estimate the probabilities and time intervals for repeatedly excessive polypharmacy, because doing so may allow for the identification of specific populations that are likely to benefit from preventive measures.

## Materials and Methods

### Data Collection

We obtained the claims datasets of a 1,000,000-person cohort (Longitudinal Health Insurance Database 2000, LHID2000) from the National Health Research Institutes in Miaoli, Taiwan, which had managed all of the archived claims of the NHI program in Taiwan through the project of the NHI Research Database^1^ until 2015. Taiwan’s NHI started as a universal health insurance program in 1995 and covered approximately 23,815,000 beneficiaries, or 98.1% of Taiwan’s entire population, as of the end of 2016 (Cheng, 2003; Bureau of National Health Insurance in Taiwan, 2017). The 1,000,000-person cohort was randomly sampled from those beneficiaries who had been insured under the NHI program from 1995 to 2000. Their claims data from 1996 to 2013 were collected together to form a longitudinal dataset for research use. Those who were born or initially insured after 2000 would not be included in these cohort datasets. Any identifying data for the patients and healthcare facilities included in the datasets have been encrypted to protect privacy.

For the analysis in this study, we used only two types of files included in the cohort datasets: ambulatory visits and prescriptions. A record in the ambulatory visit file contains the data for a single, including the patient’s identification number, date of birth, sex, date of visit, visited specialty, duration of drug supply, and three fields for diagnoses coded according to the International Classification of Diseases, Ninth Revision, Clinical Modification (ICD-9-CM). A record in the prescription file represents one prescribed drug item. Different brands, strengths, and forms of each drug are officially assigned a unique code for claims. A single visit may thus be associated with the prescriptions of several drug items, and the two types of files are linked with each other through common key fields.

The conduct of the study had been approved by the institutional review board (2013-04-005E) of Taipei Veterans General Hospital, Taipei, Taiwan.

### Study Design

Because individual beneficiaries might enter into or drop out of the NHI program at any time, our analysis was limited to those beneficiaries who still had non-emergent ambulatory visits for Western medicine after 2000, while the visits for dentistry and traditional Chinese medicine were not taken into consideration. Furthermore, the analysis also excluded those beneficiaries, foreigners in most cases, whose sex was not noted or otherwise clearly indicated in their claims files. Among the remaining beneficiaries, we then identified those who had polypharmacy for the first time after 2000 and were at least 18 years old. Polypharmacy was operationally defined as the inclusion of ten or more drug items in one prescription with a drug supply duration of more than 7 days. Within the NHI, a maximum of 7 days of drug supply can be prescribed for illness other than chronic diseases. Therefore, we effectively excluded visits made merely for acute illnesses. Furthermore, if a given drug was prescribed more than once with different directions, e.g., t.i.d. (ter in die) for one instance and h.s. p.r.n. (hora somni pro re nata) for another, it was viewed as one drug item.

For the patients with first-time excessive polypharmacy, we computed their frequency distributions in terms of sex, age, and principal diagnosis at the time of the first prescription of excessive polypharmacy. The age distribution was divided into one of three age groups: young (between 18 and 39 years old), middle-aged (between 40 and 64 years old), and old (65 years old or older). The principal diagnosis in ICD-9-CM was further reclassified into one of nearly 200 primary diagnoses adopted by the National Ambulatory Medical Care Survey (NAMCS) and National Hospital Ambulatory Medical Care Survey (NHAMCS) data in the United States (e.g., ICD-9-CM codes 430-438 were reclassified as cerebrovascular disease, 401 as essential hypertension) (Schappert and Burt, 2006).

To investigate whether and how soon excessive polypharmacy reappeared, we determined the amount of time that passed between the first and second prescriptions of excessive polypharmacy for each beneficiary with excessive polypharmacy. If a second prescription of excessive polypharmacy did not occur, we instead determined the amount of time that passed between the first prescription of excessive polypharmacy and the last ambulatory visit. That is, we used survival analysis to assess the probability of having or not having repeatedly excessive polypharmacy, where the second prescription of excessive polypharmacy was the event, the duration between the first and second prescriptions of excessive polypharmacy was the time to event, and the data were right-censored. The patient’s sex and age group were used to estimate the probabilities of repeatedly excessive polypharmacy during the full 13-year observation period.

### Qualitative Analysis

Descriptive data for the patients with first-time excessive polypharmacy occurring after 2000 and at an age of 18 years or older are presented herein. The relevant computations were undertaken with the Perl programming language (version 5.26.1.1, Perl Foundation, Walnut, CA, United States^2^). We used the Pearson’s chi-square test to compare the occurrences of repeatedly excessive polypharmacy. Furthermore, we created survival curves and estimated the distributions of repeatedly excessive polypharmacy with the Kaplan-Meier estimator. The log-rank test was used to test whether there was a difference between survival curves stratified by sex or age group. A two-sided *p* value < 0.05 was regarded as statistically significant. The statistical analyses were performed with the R software (version 3.4.2, R Foundation for Statistical Computing, Vienna, Austria^3^) and its package ‘survival’ (version 2.41-3).

## Results

In the 1,000,000-person cohort dataset covering the period from 1996 to 2013, only 928,535 people had ambulatory visits after 2000. This group consisted of 225,278 children, 355,330 young people, 263,529 middle-aged people, and 84,398 old people, and their average age at the beginning of 2001 was 33.9 ± 20.2 years old. After excluding those aged younger than 18 years old, a total of 703,257 people (349,217 women and 354,040 men) were included for further analysis.

In total, 5.36% of the people (*n* = 37,703) with ambulatory visits had excessive polypharmacy for the first time and had received one or more prescription(s) of excessive polypharmacy at an age of 18 years or older during the period from 2001 to 2013. Men (5.5%, *n* = 19,444) had a higher rate of first-time excessive polypharmacy than women (5.2%, *n* = 18,259) (χ^2^ = 24.1, *p* < 0.001). The first prescriptions of excessive polypharmacy were distributed in each year, and the average age at the first prescription of excessive polypharmacy was 64.2 ± 14.7 years old, with those receiving a first prescription of excessive polypharmacy consisting of 2,222 young people (0.6%), 15,507 middle-aged people (5.9%), and 19,974 old people (23.7%) (χ^2^ = 7200, *p* < 0.001) (**Table 1**).

**Table 1:** Frequency distribution of the first prescriptions of excessive polypharmacy, stratified by year and patient age group.

Year	Young	Middle-aged	Old	Total
2001	176	1,180	1,584	2,940
2002	183	1,326	1,783	3,292
2003	204	1,449	1,959	3,612
2004	213	1,411	1,881	3,505
2005	134	1,075	1,335	2,544
2006	149	878	1,235	2,262
2007	127	941	1,300	2,368
2008	171	991	1,324	2,486
2009	144	1,052	1,260	2,456
2010	156	1,197	1,559	2,912
2011	168	1,298	1,575	3,041
2012	223	1,398	1,683	3,304
2013	174	1,311	1,496	2,981
Total	2,222	15,507	19,974	37,703

More than three-fifths (62.3%, *n* = 23,495) of the first prescriptions of excessive polypharmacy were issued by internists, followed by family physicians (16.9%, *n* = 6,355), neurologists (8.4%, *n* = 3,166), and surgeons (4.1%, *n* = 1,544) (**Table 2**). The top five primary diagnoses most frequently seen at the first prescriptions of excessive polypharmacy were diabetes mellitus (13.6%, *n* = 5,141), essential hypertension (11.6%, *n* = 4,392), other malignant neoplasms (7.8%, *n* = 2,931), other heart disease (7.1%, *n* = 2,661), and cerebrovascular disease (6.2%, *n* = 2,322) (**Table 3**).

**Table 2:** Frequency distribution of the first prescriptions of excessive polypharmacy, stratified by physician specialty and patient age group.

Specialty	Young	Middle-aged	Old	Total (%)
Internal medicine	1,059	9,371	13,065	23,495 (62.3)
Family medicine	456	2,849	3,050	6,355 (16.9)
Neurology	76	1,031	2,059	3,166 (8.4)
Surgery	99	762	683	1,544 (4.1)
Psychiatry	222	358	112	692 (1.8)
Neurosurgery	19	136	192	347 (0.9)
Radiation oncology	27	194	120	341 (0.9)
Rehabilitation	19	130	182	331 (0.9)
Pediatrics	85	134	62	281 (0.7)
Orthopedics	12	76	167	255 (0.7)
Gynecology	39	153	49	241 (0.6)
Otolaryngology	65	131	36	232 (0.6)
Urology	10	78	102	190 (0.5)
Dermatology	27	52	24	103 (0.3)
Others	7	52	71	130 (0.3)
Total	2,222	15,507	19,974	37,703

**Table 3:** Frequency distribution of the first prescriptions of excessive polypharmacy, stratified by principal diagnosis and patient age group.

Diagnosisaaa	Young	Middle-aged	Old	Total (%)
Diabetes mellitus	118	2,417	2606	5,141 (13.6)
Essential hypertension	107	1,891	2,394	4,392 (11.6)
Other malignant neoplasms	128	1,509	1,294	2,931 (7.8)
Other heart disease	52	923	1,686	2,661 (7.1)
Cerebrovascular disease	25	717	1,580	2,322 (6.2)
Other ischemic heart disease	14	421	831	1,266 (3.4)
Other diseases of the urinary system	80	520	655	1,255 (3.3)
Asthma	134	459	450	1,043 (2.8)
Other diseases of the digestive system	114	414	295	823 (2.2)
Chronic and unspecified bronchitis	39	180	558	777 (2.1)
Other chronic obstructive pulmonary disease and allied conditions	5	114	622	741 (2.0)
Malignant neoplasm of breast	53	551	111	715 (1.9)
Other infectious and parasitic diseases	55	241	394	690 (1.8)
Malignant neoplasm of colon and rectum	27	326	337	690 (1.8)
Coronary atherosclerosis	5	216	433	654 (1.7)
Ulcer of stomach and small intestine	45	228	309	582 (1.5)
Other disorders of the central nervous system	31	131	371	533 (1.4)
Disorders of lipid metabolism	23	251	241	515 (1.4)
Others	1,167	3,998	4,807	9,972 (26.4)
Total	2,222	15,507	19,974	37,703

*aaa The diagnosis was reclassified from ICD-9-CM codes according to the NAMCS/NHAMCS data in the United States (Schappert and Burt, 2006).*

**FIGURE 1:**
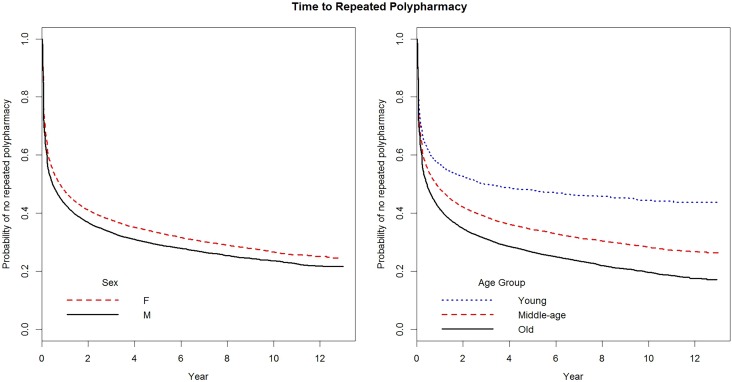
Probabilities of having no repeated prescription of excessive polypharmacy among patients, stratified by sex or age groups (both *p* < 0.001 by log-rank test), 2001–2013.

Among all the people with polypharmacy, there was an average of 6.1 ± 10.1 prescriptions of excessive polypharmacy, although 13,619 (36.1%) people received only one prescription of excessive polypharmacy. Men exhibited a slightly greater tendency to have repeated prescriptions of excessive polypharmacy than women [64.5% (12,550/19,444) vs. 63.2% (11,534/18,259), *p* < 0.001], while the disparities for repeated prescriptions of excessive polypharmacy were more striking among young people (49.6%, 1,102/2,222), middle-aged people (61.9%, 9,602/15,507), and old people (67.0%, 13,380/19,974) (*p* < 0.001). The probability of having no repeated prescription of excessive polypharmacy was 59.9% at 3 months after the first prescription, 53.6% at 6 months, 48.1% at 1 year, and 43.1% at 2 years. The median times to repeated prescription of excessive polypharmacy or the last ambulatory visit were 5.5 (95% CI: 5.3–6.1) months in men, 9.4 (8.7–10.1) months in women, 35.0 (26.2–62.4) months in young people, 10.1 (9.2–11.0) months in middle-aged people, and 5.29 (4.86–5.52) months in old people. While the respective Kaplan-Meier curves for men and women of having no repeated prescription of excessive polypharmacy ran close together, the curves for young people, middle-aged people, and old people were more widely separated (**Figure 1**). Furthermore, both the estimated survival curves of no repeatedly excessive polypharmacy between sex groups and age groups showed significant differences by log-rank test (χ^2^ = 91.7, *p* < 0.001 and χ^2^ = 432, *p* < 0.001, respectively).

## Discussion

In Taiwan from 2001 to 2013, repeated prescriptions of excessive polypharmacy (defined as a prescription with ten or more drug items in the present study) in a non-emergent visit among individuals who had already experienced first-time polypharmacy mostly occurred among specific groups: men, the elderly, patients with chronic disease(s) (i.e., diabetes mellitus, essential hypertension, heart disease, and cerebrovascular diseases) or cancer(s), and patients with visits at internists. Within half a year, half of the male patients and half of the old patients with exhibited first-time excessive polypharmacy were likely to have repeatedly excessive polypharmacy. In comparison, female patients, middle-aged patients, and young patients were less likely to have repeatedly excessive polypharmacy than their male and elderly counterparts. More than one-third of the individuals with exhibited first-time excessive polypharmacy exhibited no repeatedly excessive polypharmacy during the study period. The probability of having no repeated prescription of excessive polypharmacy decreased with time, but nearly half of the studied patients still remained excessive polypharmacy-free 1 year after their initial excessive polypharmacy.

Our results revealed that the incidence of first-time excessive polypharmacy and the occurrence of repeatedly excessive polypharmacy were slightly higher among men than women in Taiwan. Moreover, half of the men had repeatedly excessive polypharmacy sooner, that is, by nearly 4 months, than half of the women. In contrast, studies in the United States and Scotland found that women were more likely to have polypharmacy than men (Qato et al., 2008; Payne et al., 2014; Cashion et al., 2015). The difference between the rates of polypharmacy may be explained by different help-seeking behaviors among the genders in the United States and Scotland versus Taiwan. One nationwide study found that Taiwanese women tended to make ambulatory care visits more frequently than men (45.7% vs. 34.5%, ≥ 13 visits per person in a year) (Chen et al., 2006). Specifically, older Taiwanese women with higher education levels were more likely to choose multiple sources of health care (Liu and Liu, 2010). In other words, the shared number of drug items in one prescription would decrease by dividing a larger number of visits among the women in Taiwan. Therefore, caution should be used in interpreting the evidence provided in this study and making comparisons between countries with various cultural backgrounds.

Meanwhile, the patients identified as exhibiting first-time excessive polypharmacy were distributed disproportionately among different age groups, such that the incidence increased drastically with age (0.6% for young people, 7.6% for middle-aged people, and 23.67% for old people). Also, the old patients had the shortest intervals for repeatedly excessive polypharmacy, followed by the middle-aged patients then the young patients. These findings were not surprising because the risk of multi-comorbidity increases with increasing age, so does physiopathological deterioration (Milton et al., 2008). Besides aging, the medical problems of diabetes mellitus, essential hypertension, heart disease, cerebrovascular disease, and cancer were also predictors of newly developed excessive polypharmacy in Taiwan. For patients with a history of four previous chronic diseases, the prescriptions of multiple medications were aimed at achieving optimal outcomes via aggressive treatment according to evidence-based guidelines, even though clinical guidelines focused exclusively on individual conditions may increase the risk of problematic polypharmacy (Emslie-Smith et al., 2003; Guthrie et al., 2012). At the same time, for patients with cancer, for example, the prescription of multiple regimens for therapeutic effect and symptom relief would generally be deemed reasonable. The frequency distribution of specialists who prescribed patients’ first prescriptions of excessive polypharmacy was generally consistent with the health conditions that the given specialists would typically be charged with treating. Internists were particularly noteworthy in terms of prescribing multiple medications, possibly because they are relatively likely, in comparison to other types of specialists, to encounter complex health conditions, seeing the highest proportion of such cases among practicing physicians across specialties in Taiwan (20.8% for internists, 7.7% for family physicians, 2.2% for neurologists, and 8.3% for surgeons in 2013) (Taiwan Medical Association, 2014). This relatively high rate of excessive polypharmacy prescriptions from internists may also be due in part to the fact that there are no specific residency programs related to medication management for specialties other than geriatrics or family medicine in Taiwan. Nevertheless, the occurrence of polypharmacy in Taiwan was similar to the rates in other countries. In the United States, 29% of community-dwelling older adults (that is, those aged 57 to 85 years) had concurrent use of five or more prescription medications (Qato et al., 2008). In Scotland, the prevalence of polypharmacy was around one-fifth (16.9% received four to nine regular medications in 1 year, and 4.6% received ten or more medications) among adults in primary care settings according to a population-based data analysis (Payne et al., 2014). In Germany, 26.7% of the older patients (that is, those aged 70 years or more) used five or more chronically prescribed drugs (Junius-Walker et al., 2007). However, detailed comparisons of the rates of polypharmacy between countries are challenging due to discrepant definitions and various healthcare systems. In the current study, we ruled in patients with polypharmacy using a relatively strict definition, i.e., the inclusion of ten or more drug items in one prescription, and excluded patients with previously identified excessive polypharmacy.

Our most important finding was that, within the 13-year period covered by the study, most of the cases of excessive polypharmacy were not persistent, meaning they were not repeated in a short period of time (i.e., 6 months), at individual levels. To our knowledge, this is the first time that the rates of excessive polypharmacy among Taiwanese adults have been calculated and tracked longitudinally using survival analysis. One possible explanation for the above finding is that the drug utilization and help-seeking behaviors among patients might have been problem- or disease-oriented in general, rather than being medication-oriented. Since we tried to rule out acute illnesses by excluding medications prescribed with only short-term supplies, only medications for subacute and chronic conditions were generally considered. Among the one-third of the patients with only one instance of exhibited excessive polypharmacy, the need for the use of multiple medications may well have disappeared because their problems were transient. For the two-thirds of the patients with repeatedly excessive polypharmacy, the probabilities of having no excessive polypharmacy decreased sharply within 2 years after the first prescription of excessive polypharmacy. In general, patients in stable condition could receive continuous prescriptions for chronic diseases for 3 months at most from physicians in Taiwan. However, more than half of these patients did not have excessive polypharmacy within three or 6 months of the initial excessive polypharmacy, which indicated that their clinical conditions were not in need of regular use of multiple medications. Another possible explanation is that patients might seek medical help from other types of specialists after having specific health conditions were confirmed. Otherwise, some patients with a newly diagnosed or worsening chronic disease might have experienced unsatisfactory therapeutic effects (including intolerable side effects) after increasing their medication regimens. In any case, it can be concluded that, in general, long-term concurrent use of ten or more medications was not a “necessity” for most patients.

Furthermore, the rates of first-time excessive polypharmacy among patients changed from year to year and there were no consistent trends noted. From 2012 to 2013, the policy for setting the threshold of medications per prescription seemed to have a limited effect on the development of excessive polypharmacy because the overall rate was still higher than in previous years, i.e., from 2005 to 2010. This finding may be explained by delayed or ineffective interventions taken by the healthcare facilities in order to tackle the issue of polypharmacy, at least within a relatively short period of time; however, the long-term effects of such policies should be evaluated further. Another possible explanation is that the help-seeking behaviors and perceived need for medical treatment among patients may remain unchanged if there is no effective deprescribing intervention being applied to the system. However, the policy seeking to limit polypharmacy also raise some potential concerns. First, it does not differentiate between appropriate and inappropriate prescribing, or indicate quality of care, and a crudely enacted decrease in the prescription of drug items may raise the risks of undertreatment and underprescription (Kuijpers et al., 2008), as well as the risk of medication-related disagreements between doctors and patients (Junius-Walker et al., 2007). Secondly, the policy seeks to limit the concurrent use of multiple medications for transient needs, which may result in fragmentation of care and overall polypharmacy. For instance, patients could engage in “doctor shopping,” i.e., frequent visits to and switching of physicians, in order to meet their medical needs because co-payments are cheap and there is no existing limitation set on access to ambulatory care (including referrals) under the current NHI program in Taiwan (Chen et al., 2006). Another study showed that patients in Taiwan would increase their medical visits to various sites in order to acquire more zolpidem pills despite the government setting related prescribing limitations (i.e., frequency and amount) for physicians (Lu et al., 2015). Therefore, we propose that it may not be necessary to restrict drug items per prescription across the general public. Instead, comprehensive deprescribing interventions should be applied to target patients (such as male patients and old patients) or physicians (such as internists) in high-risk groups for utilizing or prescribing problematic polypharmacy. Evidence has proved that approaches such as shared decision making between patients and healthcare providers; providing infrastructural support for the coordination and continuity of care (Bemben, 2016), especially for multi-morbidity patients (Duerden et al., 2013); and educational programs on deprescribing for physicians are effective in improving problematic polypharmacy.

In the current study, we mainly examined the patterns of excessive polypharmacy, the probability of having repeatedly excessive polypharmacy and its association with time, and their related factors. The classification of medications, prescribing appropriateness as measured by explicit tools (e.g., Beers’ criteria) (Cooper et al., 2015), and the outcomes of polypharmacy were beyond the scope of our study. The retrospective sample data also did not include data regarding some variables that would affect the use of drugs, such as educational levels (Qato et al., 2008), so potential confounding factors could not be controlled. Data regarding certain demographic factors, such as individuals’ economic statuses (Payne et al., 2014) or geographic variations (Cashion et al., 2015) that had been considered associated factors for polypharmacy were also not considered in the current study. Moreover, patients with various health conditions or disease severities in their subsequent ambulatory visits, emergency department visits, and hospitalizations or deaths were not considered. In addition, our study also had some limitations that might lead to the underestimation of the instances of excessive polypharmacy. Firstly, we excluded prescriptions with drug supply durations of 7 days or less in order to only consider prescriptions for chronic conditions, but those short-term medications aimed at managing chronic diseases were not identified in detail. Secondly, data on over-the-counter medicines or dietary supplements which were not covered by the NHI program were not included in the database. Thirdly, we did not consider the prescriptions from visits to dentists or traditional Chinese medicine practices. Collective polypharmacy resulting from having prescriptions from visits to various physicians at one time was also beyond the scope of this study. Further analysis of the relationship between persistently excessive polypharmacy and the presence of comorbidities should be explored in future study. In addition, we studied the patterns of excessive polypharmacy among patients with first-time excessive polypharmacy, so information regarding patients with previously existing excessive polypharmacy was not considered.

Despite these limitations, our study demonstrates both the transient and persistent trends of excessive polypharmacy among certain groups and their associations with time. The findings of the present research provide a systemic understanding of excessive polypharmacy and its changes in terms of prescription/utilization over time. For policymakers, as well as for clinicians, it is important to follow the trends in polypharmacy and to tailor deprescribing measures on an individual level according to patients’ needs and risks. Our results can contribute to the modification of healthcare policy and the research and development of medication management to improve the prescribing of polypharmacy.

## Conclusion

Our findings indicate that the occurrence of excessive polypharmacy was transient and would not re-appear within 6 months among most patients, with the exception of men and old people. Instead of applying coercive policies regarding the maximum number of medications a physician can prescribe to all patients, regardless of their needs, tailored deprescribing interventions should be considered for targeted application among identifiable high-risk groups.

## Author Contributions

Y-JW, P-CL, and T-JC conceived and designed the study. Y-JW, S-CC, and T-JC analyzed the data. S-CC, L-FC, and T-JC contributed analysis tools. Y-CChe, and Y-CCho reviewed drafts of the paper. Y-JW wrote the paper. All authors read and approved the final manuscript.

## Conflict of Interest Statement

The authors declare that the research was conducted in the absence of any commercial or financial relationships that could be construed as a potential conflict of interest.

## Footnotes

1 https://nhird.nhri.org.tw/

2 https://www.perl.org/

3 https://www.r-project.org/
